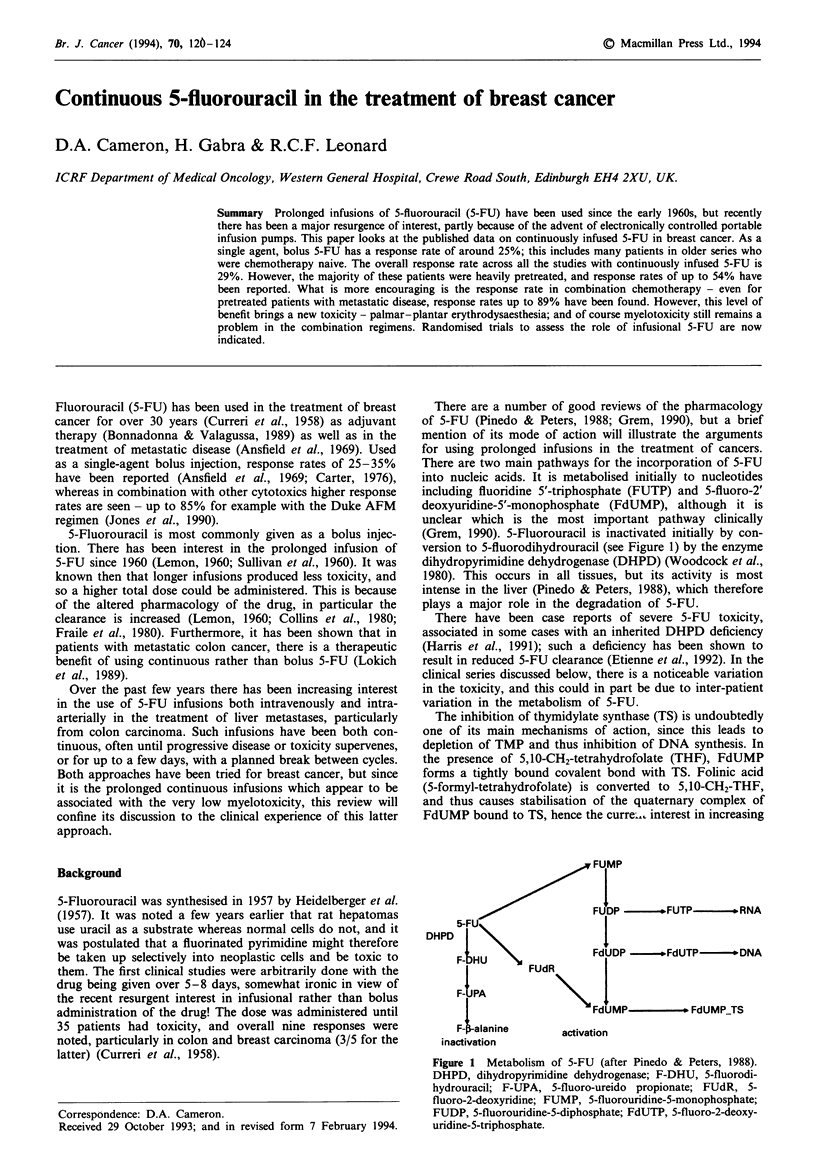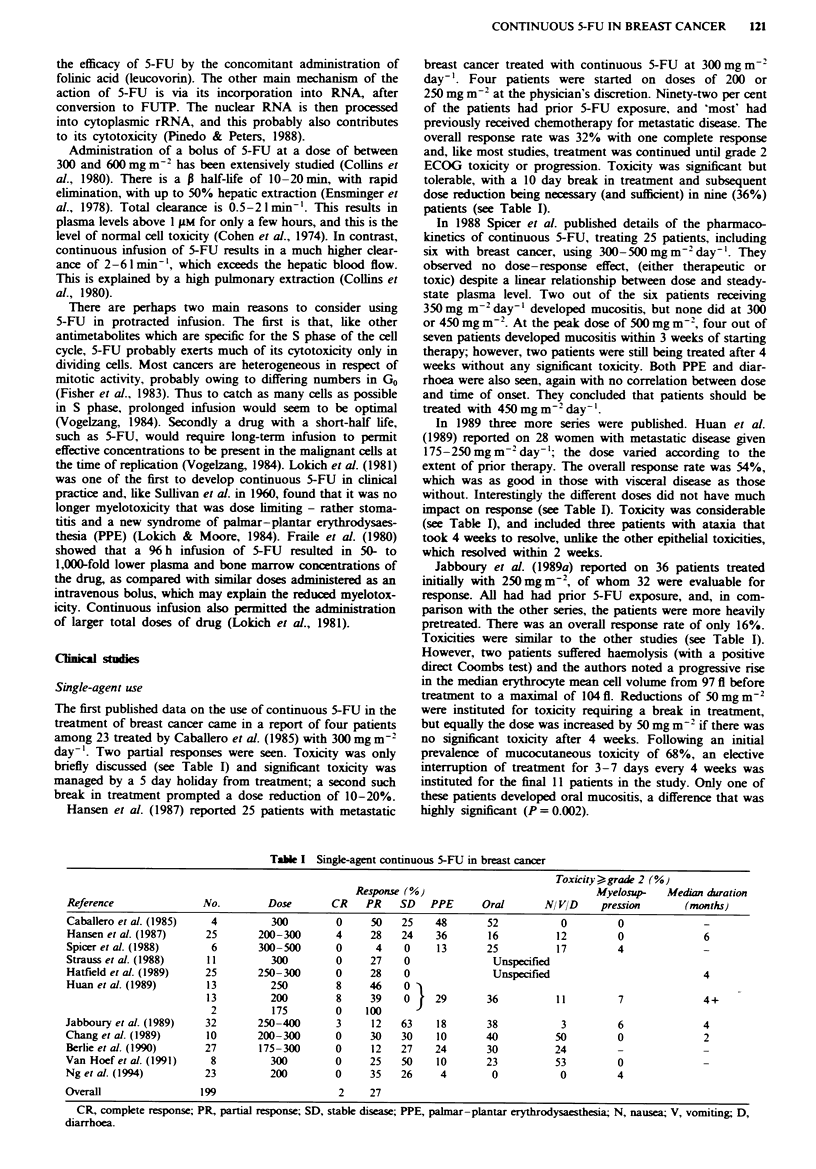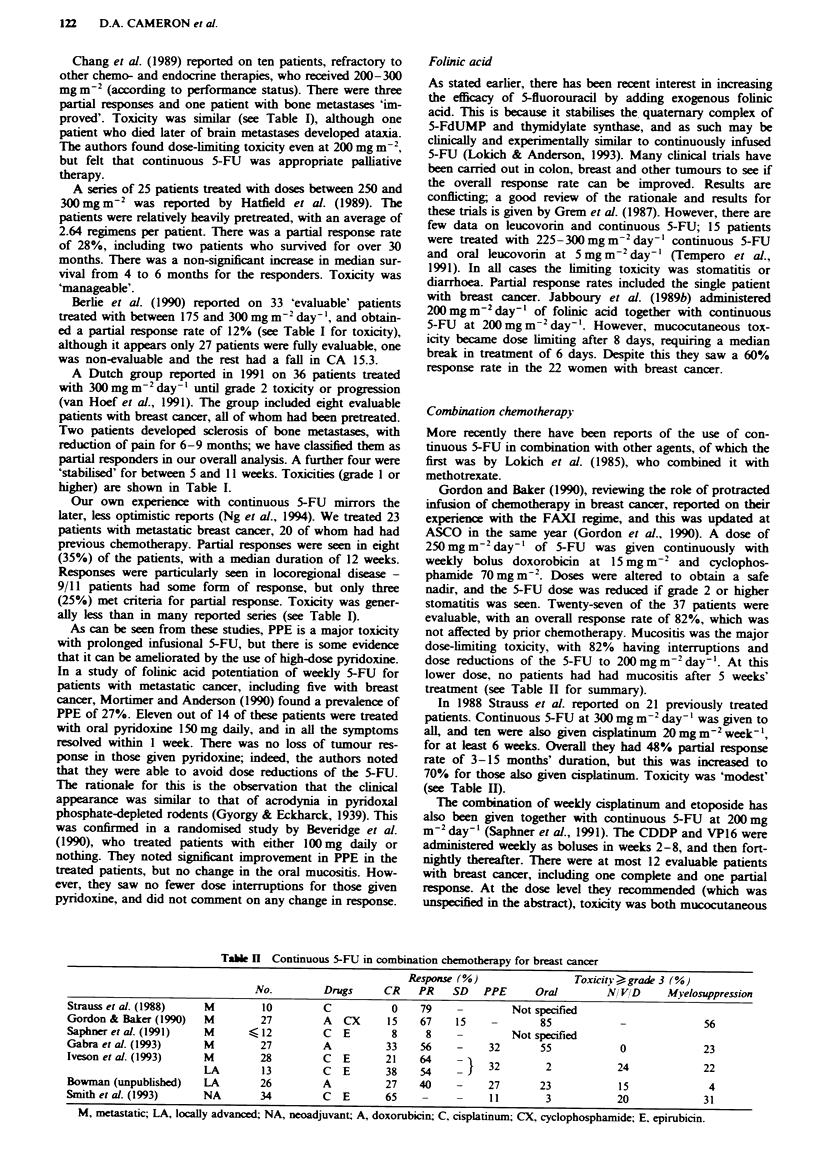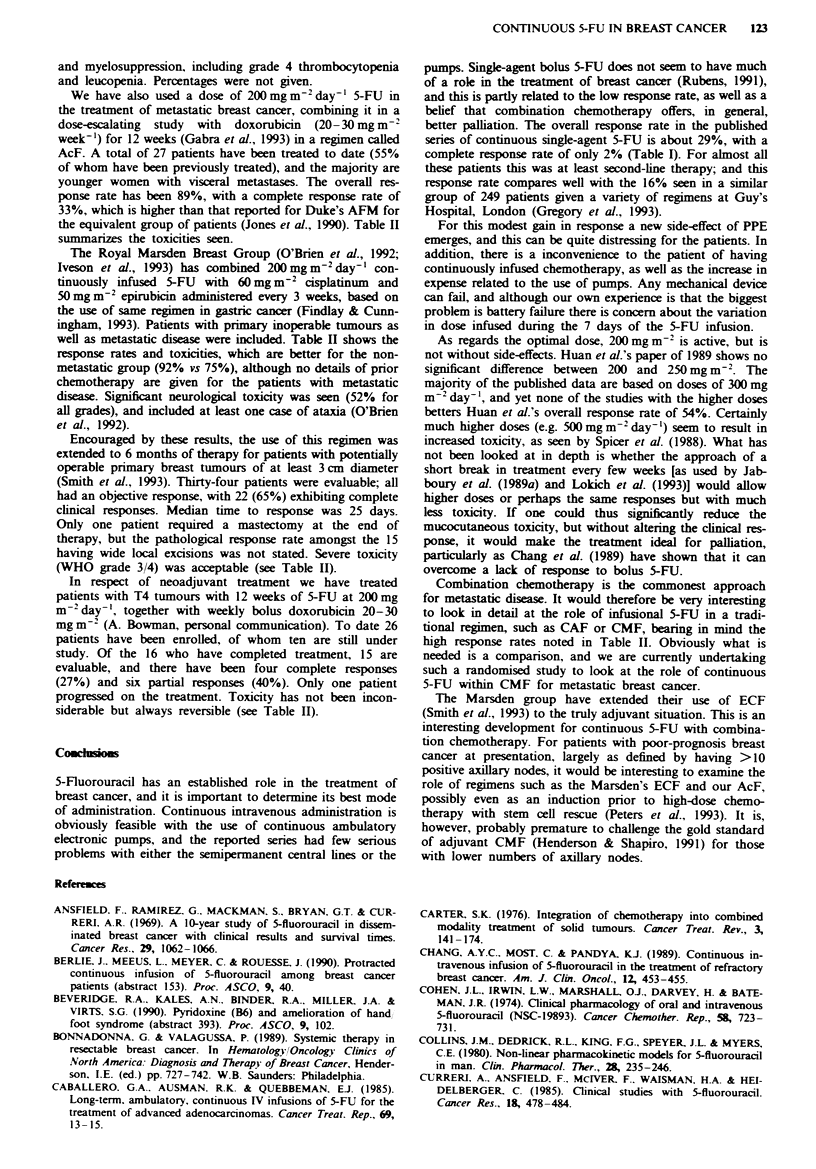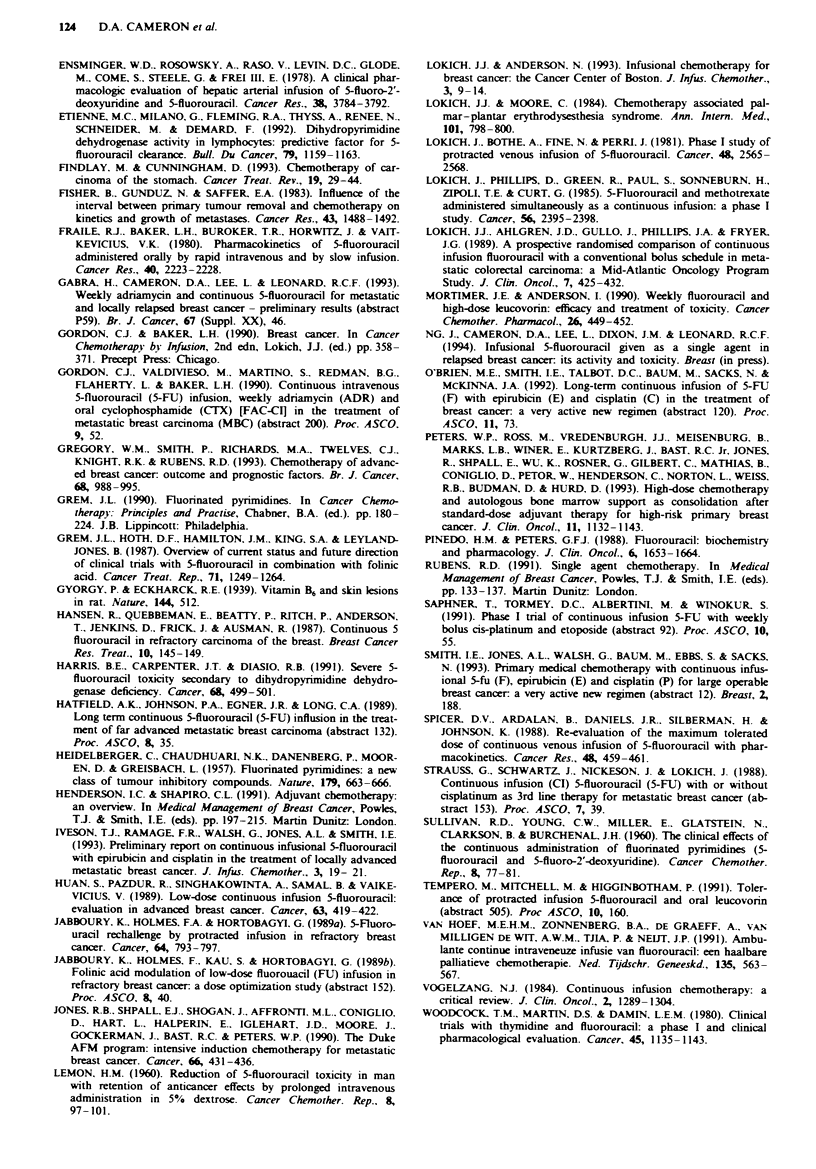# Continuous 5-fluorouracil in the treatment of breast cancer.

**DOI:** 10.1038/bjc.1994.259

**Published:** 1994-07

**Authors:** D. A. Cameron, H. Gabra, R. C. Leonard

**Affiliations:** ICRF Department of Medical Oncology, Western General Hospital, Edinburgh, UK.

## Abstract

Prolonged infusions of 5-fluorouracil (5-FU) have been used since the early 1960s, but recently there has been a major resurgence of interest, partly because of the advent of electronically controlled portable infusion pumps. This paper looks at the published data on continuously infused 5-FU in breast cancer. As a single agent, bolus 5-FU has a response rate of around 25%; this includes many patients in older series who were chemotherapy naive. The overall response rate across all the studies with continuously infused 5-FU is 29%. However, the majority of these patients were heavily pretreated, and response rates of up to 54% have been reported. What is more encouraging is the response rate in combination chemotherapy--even for pretreated patients with metastatic disease, response rates up to 89% have been found. However, this level of benefit brings a new toxicity--palmar--plantar erythrodysaesthesia; and of course myelotoxicity still remains a problem in the combination regimens. Randomised trials to assess the role of infusional 5-FU are now indicated.


 © Macmillan Press Ltd., 1994

### Continuous 5-fluorouracil in the treatment of breast cancer

D.A. Cameron, H. Gabra & R.C.F. Leonard

*ICRF Department of Medical Oncology, Western General Hospital, Crewe Road South, Edinburgh EH4 2XU, UK.*

**Summary** Prolonged infusions of 5-fluorouracil (5-FU) have been used since the early 1960s, but recently there has been a major resurgence of interest, partly because of the advent of electronically controlled portable infusion pumps. This paper looks at the published data on continuously infused 5-FU in breast cancer. As a single agent, bolus 5-FU has a response rate of around 25%; this includes many patients in older series who were chemotherapy naive. The overall response rate across all the studies with continuously infused 5-FU is 29%. However, the majority of these patients were heavily pretreated, and response rates of up to 54% have been reported. What is more encouraging is the response rate in combination chemotherapy – even for pretreated patients with metastatic disease, response rates up to 89% have been found. However, this level of benefit brings a new toxicity – palmar–plantar erythrodysaesthesia; and of course myelotoxicity still remains a problem in the combination regimens. Randomised trials to assess the role of infusional 5-FU are now indicated.

Fluorouracil (5-FU) has been used in the treatment of breast cancer for over 30 years (Curreri *et al.*, 1958) as adjuvant therapy (Bonnadonna & Valagussa, 1989) as well as in the treatment of metastatic disease (Ansfield *et al.*, 1969). Used as a single-agent bolus injection, response rates of 25–35% have been reported (Ansfield *et al.*, 1969; Carter, 1976), whereas in combination with other cytotoxics higher response rates are seen – up to 85% for example with the Duke AFM regimen (Jones *et al.*, 1990).

5-Fluorouracil is most commonly given as a bolus injection. There has been interest in the prolonged infusion of 5-FU since 1960 (Lemon, 1960; Sullivan *et al.*, 1960). It was known then that longer infusions produced less toxicity, and so a higher total dose could be administered. This is because of the altered pharmacology of the drug, in particular the clearance is increased (Lemon, 1960; Collins *et al.*, 1980; Fraile *et al.*, 1980). Furthermore, it has been shown that in patients with metastatic colon cancer, there is a therapeutic benefit of using continuous rather than bolus 5-FU (Lokich *et al.*, 1989).

Over the past few years there has been increasing interest in the use of 5-FU infusions both intravenously and intra-arterially in the treatment of liver metastases, particularly from colon carcinoma. Such infusions have been both continuous, often until progressive disease or toxicity supervenes, or for up to a few days, with a planned break between cycles. Both approaches have been tried for breast cancer, but since it is the prolonged continuous infusions which appear to be associated with the very low myelotoxicity, this review will confine its discussion to the clinical experience of this latter approach.

### Background

5-Fluorouracil was synthesised in 1957 by Heidelberger *et al.* (1957). It was noted a few years earlier that rat hepatomas use uracil as a substrate whereas normal cells do not, and it was postulated that a fluorinated pyrimidine might therefore be taken up selectively into neoplastic cells and be toxic to them. The first clinical studies were arbitrarily done with the drug being given over 5–8 days, somewhat ironic in view of the recent resurgent interest in infusional rather than bolus administration of the drug! The dose was administered until 35 patients had toxicity, and overall nine responses were noted, particularly in colon and breast carcinoma (3/5 for the latter) (Curreri *et al.*, 1958).

Correspondence: D.A. Cameron.
Received 29 October 1993; and in revised form 7 February 1994.

There are a number of good reviews of the pharmacology of 5-FU (Pinedo & Peters, 1988; Grem, 1990), but a brief mention of its mode of action will illustrate the arguments for using prolonged infusions in the treatment of cancers. There are two main pathways for the incorporation of 5-FU into nucleic acids. It is metabolised initially to nucleotides including fluoridine 5′-triphosphate (FUTP) and 5-fluoro-2′ deoxyuridine-5′-monophosphate (FdUMP), although it is unclear which is the most important pathway clinically (Grem, 1990). 5-Fluorouracil is inactivated initially by conversion to 5-fluorodihydrouracil (see Figure 1) by the enzyme dihydropyrimidine dehydrogenase (DHPD) (Woodcock *et al.*, 1980). This occurs in all tissues, but its activity is most intense in the liver (Pinedo & Peters, 1988), which therefore plays a major role in the degradation of 5-FU.

There have been case reports of severe 5-FU toxicity, associated in some cases with an inherited DHPD deficiency (Harris *et al.*, 1991); such a deficiency has been shown to result in reduced 5-FU clearance (Etienne *et al.*, 1992). In the clinical series discussed below, there is a noticeable variation in the toxicity, and this could in part be due to inter-patient variation in the metabolism of 5-FU.

The inhibition of thymidylate synthase (TS) is undoubtedly one of its main mechanisms of action, since this leads to depletion of TMP and thus inhibition of DNA synthesis. In the presence of 5,10-$CH_2$-tetrahydrofolate (THF), FdUMP forms a tightly bound covalent bond with TS. Folinic acid (5-formyl-tetrahydrofolate) is converted to 5,10-$CH_2$-THF, and thus causes stabilisation of the quaternary complex of FdUMP bound to TS, hence the current interest in increasing

**Figure 1** Metabolism of 5-FU (after Pinedo & Peters, 1988). DHPD, dihydropyrimidine dehydrogenase; F-DHU, 5-fluorodihydrouracil; F-UPA, 5-fluoro-ureido propionate; FUdR, 5-fluoro-2-deoxyridine; FUMP, 5-fluorouridine-5-monophosphate; FUDP, 5-fluorouridine-5-diphosphate; FdUTP, 5-fluoro-2-deoxyuridine-5-triphosphate.

the efficacy of 5-FU by the concomitant administration of folinic acid (leucovorin). The other main mechanism of the action of 5-FU is via its incorporation into RNA, after conversion to FUTP. The nuclear RNA is then processed into cytoplasmic rRNA, and this probably also contributes to its cytotoxicity (Pinedo & Peters, 1988).

Administration of a bolus of 5-FU at a dose of between 300 and 600 mg m$^{-2}$ has been extensively studied (Collins *et al.*, 1980). There is a $\beta$ half-life of 10–20 min, with rapid elimination, with up to 50% hepatic extraction (Ensminger *et al.*, 1978). Total clearance is 0.5–2 l min$^{-1}$. This results in plasma levels above 1 μM for only a few hours, and this is the level of normal cell toxicity (Cohen *et al.*, 1974). In contrast, continuous infusion of 5-FU results in a much higher clearance of 2–6 l min$^{-1}$, which exceeds the hepatic blood flow. This is explained by a high pulmonary extraction (Collins *et al.*, 1980).

There are perhaps two main reasons to consider using 5-FU in protracted infusion. The first is that, like other antimetabolites which are specific for the S phase of the cell cycle, 5-FU probably exerts much of its cytotoxicity only in dividing cells. Most cancers are heterogeneous in respect of mitotic activity, probably owing to differing numbers in $G_0$ (Fisher *et al.*, 1983). Thus to catch as many cells as possible in S phase, prolonged infusion would seem to be optimal (Vogelzang, 1984). Secondly a drug with a short-half life, such as 5-FU, would require long-term infusion to permit effective concentrations to be present in the malignant cells at the time of replication (Vogelzang, 1984). Lokich *et al.* (1981) was one of the first to develop continuous 5-FU in clinical practice and, like Sullivan *et al.* in 1960, found that it was no longer myelotoxicity that was dose limiting – rather stomatitis and a new syndrome of palmar–plantar erythrodysaesthesia (PPE) (Lokich & Moore, 1984). Fraile *et al.* (1980) showed that a 96 h infusion of 5-FU resulted in 50- to 1,000-fold lower plasma and bone marrow concentrations of the drug, as compared with similar doses administered as an intravenous bolus, which may explain the reduced myelotoxicity. Continuous infusion also permitted the administration of larger total doses of drug (Lokich *et al.*, 1981).

### Clinical studies

### *Single-agent use*

The first published data on the use of continuous 5-FU in the treatment of breast cancer came in a report of four patients among 23 treated by Caballero *et al.* (1985) with 300 mg m$^{-2}$ day$^{-1}$. Two partial responses were seen. Toxicity was only briefly discussed (see Table I) and significant toxicity was managed by a 5 day holiday from treatment; a second such break in treatment prompted a dose reduction of 10–20%.

Hansen *et al.* (1987) reported 25 patients with metastatic breast cancer treated with continuous 5-FU at 300 mg m$^{-2}$ day$^{-1}$. Four patients were started on doses of 200 or 250 mg m$^{-2}$ at the physician's discretion. Ninety-two per cent of the patients had prior 5-FU exposure, and 'most' had previously received chemotherapy for metastatic disease. The overall response rate was 32% with one complete response and, like most studies, treatment was continued until grade 2 ECOG toxicity or progression. Toxicity was significant but tolerable, with a 10 day break in treatment and subsequent dose reduction being necessary (and sufficient) in nine (36%) patients (see Table I).

In 1988 Spicer *et al.* published details of the pharmacokinetics of continuous 5-FU, treating 25 patients, including six with breast cancer, using 300–500 mg m$^{-2}$ day$^{-1}$. They observed no dose–response effect, (either therapeutic or toxic) despite a linear relationship between dose and steady-state plasma level. Two out of the six patients receiving 350 mg m$^{-2}$ day$^{-1}$ developed mucositis, but none did at 300 or 450 mg m$^{-2}$. At the peak dose of 500 mg m$^{-2}$, four out of seven patients developed mucositis within 3 weeks of starting therapy; however, two patients were still being treated after 4 weeks without any significant toxicity. Both PPE and diarrhoea were also seen, again with no correlation between dose and time of onset. They concluded that patients should be treated with 450 mg m$^{-2}$ day$^{-1}$.

In 1989 three more series were published. Huan *et al.* (1989) reported on 28 women with metastatic disease given 175–250 mg m$^{-2}$ day$^{-1}$; the dose varied according to the extent of prior therapy. The overall response rate was 54%, which was as good in those with visceral disease as those without. Interestingly the different doses did not have much impact on response (see Table I). Toxicity was considerable (see Table I), and included three patients with ataxia that took 4 weeks to resolve, unlike the other epithelial toxicities, which resolved within 2 weeks.

Jabboury *et al.* (1989*a*) reported on 36 patients treated initially with 250 mg m$^{-2}$, of whom 32 were evaluable for response. All had had prior 5-FU exposure, and, in comparison with the other series, the patients were more heavily pretreated. There was an overall response rate of only 16%. Toxicities were similar to the other studies (see Table I). However, two patients suffered haemolysis (with a positive direct Coombs test) and the authors noted a progressive rise in the median erythrocyte mean cell volume from 97 fl before treatment to a maximal of 104 fl. Reductions of 50 mg m$^{-2}$ were instituted for toxicity requiring a break in treatment, but equally the dose was increased by 50 mg m$^{-2}$ if there was no significant toxicity after 4 weeks. Following an initial prevalence of mucocutaneous toxicity of 68%, an elective interruption of treatment for 3–7 days every 4 weeks was instituted for the final 11 patients in the study. Only one of these patients developed oral mucositis, a difference that was highly significant ($P = 0.002$).

**Table I** Single-agent continuous 5-FU in breast cancer

| Reference | No. | Dose | Response (%) CR | PR | SD | Toxicity ≥ grade 2 (%) PPE | Oral | N/V/D | Myelosuppression | Median duration (months) |
|---|---|---|---|---|---|---|---|---|---|---|
| Caballero *et al.* (1985) | 4 | 300 | 0 | 50 | 25 | 48 | 52 | 0 | 0 | – |
| Hansen *et al.* (1987) | 25 | 200–300 | 4 | 28 | 24 | 36 | 16 | 12 | 0 | 6 |
| Spicer *et al.* (1988) | 6 | 300–500 | 0 | 4 | 0 | 13 | 25 | 17 | 4 | – |
| Strauss *et al.* (1988) | 11 | 300 | 0 | 27 | 0 | | Unspecified | | | |
| Hatfield *et al.* (1989) | 25 | 250–300 | 0 | 28 | 0 | | Unspecified | | | 4 |
| Huan *et al.* (1989) | 13 | 250 | 8 | 46 | 0 | | | | | |
| | 13 | 200 | 8 | 39 | 0 | 29 | 36 | 11 | 7 | 4+ |
| | 2 | 175 | 0 | 100 | | | | | | |
| Jabboury *et al.* (1989) | 32 | 250–400 | 3 | 12 | 63 | 18 | 38 | 3 | 6 | 4 |
| Chang *et al.* (1989) | 10 | 200–300 | 0 | 30 | 30 | 10 | 40 | 50 | 0 | 2 |
| Berlie *et al.* (1990) | 27 | 175–300 | 0 | 12 | 27 | 24 | 30 | 24 | – | – |
| Van Hoef *et al.* (1991) | 8 | 300 | 0 | 25 | 50 | 10 | 23 | 53 | 0 | – |
| Ng *et al.* (1994) | 23 | 200 | 0 | 35 | 26 | 4 | 0 | 0 | 4 | |
| Overall | 199 | | 2 | 27 | | | | | | |

CR, complete response; PR, partial response; SD, stable disease; PPE, palmar–plantar erythrodysaesthesia; N, nausea; V, vomiting; D, diarrhoea.

Chang *et al.* (1989) reported on ten patients, refractory to other chemo- and endocrine therapies, who received 200–300 mg m$^{-2}$ (according to performance status). There were three partial responses and one patient with bone metastases 'improved'. Toxicity was similar (see Table I), although one patient who died later of brain metastases developed ataxia. The authors found dose-limiting toxicity even at 200 mg m$^{-2}$, but felt that continuous 5-FU was appropriate palliative therapy.

A series of 25 patients treated with doses between 250 and 300 mg m$^{-2}$ was reported by Hatfield *et al.* (1989). The patients were relatively heavily pretreated, with an average of 2.64 regimens per patient. There was a partial response rate of 28%, including two patients who survived for over 30 months. There was a non-significant increase in median survival from 4 to 6 months for the responders. Toxicity was 'manageable'.

Berlie *et al.* (1990) reported on 33 'evaluable' patients treated with between 175 and 300 mg m$^{-2}$ day$^{-1}$, and obtained a partial response rate of 12% (see Table I for toxicity), although it appears only 27 patients were fully evaluable, one was non-evaluable and the rest had a fall in CA 15.3.

A Dutch group reported in 1991 on 36 patients treated with 300 mg m$^{-2}$ day$^{-1}$ until grade 2 toxicity or progression (van Hoef *et al.*, 1991). The group included eight evaluable patients with breast cancer, all of whom had been pretreated. Two patients developed sclerosis of bone metastases, with reduction of pain for 6–9 months; we have classified them as partial responders in our overall analysis. A further four were 'stabilised' for between 5 and 11 weeks. Toxicities (grade 1 or higher) are shown in Table I.

Our own experience with continuous 5-FU mirrors the later, less optimistic reports (Ng *et al.*, 1994). We treated 23 patients with metastatic breast cancer, 20 of whom had had previous chemotherapy. Partial responses were seen in eight (35%) of the patients, with a median duration of 12 weeks. Responses were particularly seen in locoregional disease – 9/11 patients had some form of response, but only three (25%) met criteria for partial response. Toxicity was generally less than in many reported series (see Table I).

As can be seen from these studies, PPE is a major toxicity with prolonged infusional 5-FU, but there is some evidence that it can be ameliorated by the use of high-dose pyridoxine. In a study of folinic acid potentiation of weekly 5-FU for patients with metastatic cancer, including five with breast cancer, Mortimer and Anderson (1990) found a prevalence of PPE of 27%. Eleven out of 14 of these patients were treated with oral pyridoxine 150 mg daily, and in all the symptoms resolved within 1 week. There was no loss of tumour response in those given pyridoxine; indeed, the authors noted that they were able to avoid dose reductions of the 5-FU. The rationale for this is the observation that the clinical appearance was similar to that of acrodynia in pyridoxal phosphate-depleted rodents (Gyorgy & Eckharck, 1939). This was confirmed in a randomised study by Beveridge *et al.* (1990), who treated patients with either 100 mg daily or nothing. They noted significant improvement in PPE in the treated patients, but no change in the oral mucositis. However, they saw no fewer dose interruptions for those given pyridoxine, and did not comment on any change in response.

*Folinic acid*

As stated earlier, there has been recent interest in increasing the efficacy of 5-fluorouracil by adding exogenous folinic acid. This is because it stabilises the quaternary complex of 5-FdUMP and thymidylate synthase, and as such may be clinically and experimentally similar to continuously infused 5-FU (Lokich & Anderson, 1993). Many clinical trials have been carried out in colon, breast and other tumours to see if the overall response rate can be improved. Results are conflicting; a good review of the rationale and results for these trials is given by Grem *et al.* (1987). However, there are few data on leucovorin and continuous 5-FU; 15 patients were treated with 225–300 mg m$^{-2}$ day$^{-1}$ continuous 5-FU and oral leucovorin at 5 mg m$^{-2}$ day$^{-1}$ (Tempero *et al.*, 1991). In all cases the limiting toxicity was stomatitis or diarrhoea. Partial response rates included the single patient with breast cancer. Jabboury *et al.* (1989*b*) administered 200 mg m$^{-2}$ day$^{-1}$ of folinic acid together with continuous 5-FU at 200 mg m$^{-2}$ day$^{-1}$. However, mucocutaneous toxicity became dose limiting after 8 days, requiring a median break in treatment of 6 days. Despite this they saw a 60% response rate in the 22 women with breast cancer.

*Combination chemotherapy*

More recently there have been reports of the use of continuous 5-FU in combination with other agents, of which the first was by Lokich *et al.* (1985), who combined it with methotrexate.

Gordon and Baker (1990), reviewing the role of protracted infusion of chemotherapy in breast cancer, reported on their experience with the FAXI regime, and this was updated at ASCO in the same year (Gordon *et al.*, 1990). A dose of 250 mg m$^{-2}$ day$^{-1}$ of 5-FU was given continuously with weekly bolus doxorobicin at 15 mg m$^{-2}$ and cyclophosphamide 70 mg m$^{-2}$. Doses were altered to obtain a safe nadir, and the 5-FU dose was reduced if grade 2 or higher stomatitis was seen. Twenty-seven of the 37 patients were evaluable, with an overall response rate of 82%, which was not affected by prior chemotherapy. Mucositis was the major dose-limiting toxicity, with 82% having interruptions and dose reductions of the 5-FU to 200 mg m$^{-2}$ day$^{-1}$. At this lower dose, no patients had had mucositis after 5 weeks' treatment (see Table II for summary).

In 1988 Strauss *et al.* reported on 21 previously treated patients. Continuous 5-FU at 300 mg m$^{-2}$ day$^{-1}$ was given to all, and ten were also given cisplatinum 20 mg m$^{-2}$ week$^{-1}$, for at least 6 weeks. Overall they had 48% partial response rate of 3–15 months' duration, but this was increased to 70% for those also given cisplatinum. Toxicity was 'modest' (see Table II).

The combination of weekly cisplatinum and etoposide has also been given together with continuous 5-FU at 200 mg m$^{-2}$ day$^{-1}$ (Saphner *et al.*, 1991). The CDDP and VP16 were administered weekly as boluses in weeks 2–8, and then fortnightly thereafter. There were at most 12 evaluable patients with breast cancer, including one complete and one partial response. At the dose level they recommended (which was unspecified in the abstract), toxicity was both mucocutaneous

**Table II** Continuous 5-FU in combination chemotherapy for breast cancer

| | | | | Response (%) | | | | Toxicity ≥ grade 3 (%) | | |
|---|---|---|---|---|---|---|---|---|---|---|
| | | *No.* | *Drugs* | *CR* | *PR* | *SD* | *PPE* | *Oral* | *N/V/D* | *Myelosuppression* |
| Strauss *et al.* (1988) | M | 10 | C | 0 | 79 | – | | Not specified | | |
| Gordon & Baker (1990) | M | 27 | A CX | 15 | 67 | 15 | – | 85 | – | 56 |
| Saphner *et al.* (1991) | M | ≤12 | C E | 8 | 8 | – | | Not specified | | |
| Gabra *et al.* (1993) | M | 27 | A | 33 | 56 | – | 32 | 55 | 0 | 23 |
| Iveson *et al.* (1993) | M | 28 | C E | 21 | 64 | – } | 32 | 2 | 24 | 22 |
| | LA | 13 | C E | 38 | 54 | – } | | | | |
| Bowman (unpublished) | LA | 26 | A | 27 | 40 | – | 27 | 23 | 15 | 4 |
| Smith *et al.* (1993) | NA | 34 | C E | 65 | – | – | 11 | 3 | 20 | 31 |

M, metastatic; LA, locally advanced; NA, neoadjuvant; A, doxorubicin; C, cisplatinum; CX, cyclophosphamide; E, epirubicin.

and myelosuppression, including grade 4 thrombocytopenia and leucopenia. Percentages were not given.

We have also used a dose of 200 mg m$^{-2}$ day$^{-1}$ 5-FU in the treatment of metastatic breast cancer, combining it in a dose-escalating study with doxorubicin (20–30 mg m$^{-2}$ week$^{-1}$) for 12 weeks (Gabra *et al.*, 1993) in a regimen called AcF. A total of 27 patients have been treated to date (55% of whom have been previously treated), and the majority are younger women with visceral metastases. The overall response rate has been 89%, with a complete response rate of 33%, which is higher than that reported for Duke's AFM for the equivalent group of patients (Jones *et al.*, 1990). Table II summarizes the toxicities seen.

The Royal Marsden Breast Group (O'Brien *et al.*, 1992; Iveson *et al.*, 1993) has combined 200 mg m$^{-2}$ day$^{-1}$ continuously infused 5-FU with 60 mg m$^{-2}$ cisplatinum and 50 mg m$^{-2}$ epirubicin administered every 3 weeks, based on the use of same regimen in gastric cancer (Findlay & Cunningham, 1993). Patients with primary inoperable tumours as well as metastatic disease were included. Table II shows the response rates and toxicities, which are better for the non-metastatic group (92% *vs* 75%), although no details of prior chemotherapy are given for the patients with metastatic disease. Significant neurological toxicity was seen (52% for all grades), and included at least one case of ataxia (O'Brien *et al.*, 1992).

Encouraged by these results, the use of this regimen was extended to 6 months of therapy for patients with potentially operable primary breast tumours of at least 3 cm diameter (Smith *et al.*, 1993). Thirty-four patients were evaluable; all had an objective response, with 22 (65%) exhibiting complete clinical responses. Median time to response was 25 days. Only one patient required a mastectomy at the end of therapy, but the pathological response rate amongst the 15 having wide local excisions was not stated. Severe toxicity (WHO grade 3/4) was acceptable (see Table II).

In respect of neoadjuvant treatment we have treated patients with T4 tumours with 12 weeks of 5-FU at 200 mg m$^{-2}$ day$^{-1}$, together with weekly bolus doxorubicin 20–30 mg m$^{-2}$ (A. Bowman, personal communication). To date 26 patients have been enrolled, of whom ten are still under study. Of the 16 who have completed treatment, 15 are evaluable, and there have been four complete responses (27%) and six partial responses (40%). Only one patient progressed on the treatment. Toxicity has not been inconsiderable but always reversible (see Table II).

### Conclusions

5-Fluorouracil has an established role in the treatment of breast cancer, and it is important to determine its best mode of administration. Continuous intravenous administration is obviously feasible with the use of continuous ambulatory electronic pumps, and the reported series had few serious problems with either the semipermanent central lines or the pumps. Single-agent bolus 5-FU does not seem to have much of a role in the treatment of breast cancer (Rubens, 1991), and this is partly related to the low response rate, as well as a belief that combination chemotherapy offers, in general, better palliation. The overall response rate in the published series of continuous single-agent 5-FU is about 29%, with a complete response rate of only 2% (Table I). For almost all these patients this was at least second-line therapy; and this response rate compares well with the 16% seen in a similar group of 249 patients given a variety of regimens at Guy's Hospital, London (Gregory *et al.*, 1993).

For this modest gain in response a new side-effect of PPE emerges, and this can be quite distressing for the patients. In addition, there is a inconvenience to the patient of having continuously infused chemotherapy, as well as the increase in expense related to the use of pumps. Any mechanical device can fail, and although our own experience is that the biggest problem is battery failure there is concern about the variation in dose infused during the 7 days of the 5-FU infusion.

As regards the optimal dose, 200 mg m$^{-2}$ is active, but is not without side-effects. Huan *et al.*'s paper of 1989 shows no significant difference between 200 and 250 mg m$^{-2}$. The majority of the published data are based on doses of 300 mg m$^{-2}$ day$^{-1}$, and yet none of the studies with the higher doses betters Huan *et al.*'s overall response rate of 54%. Certainly much higher doses (e.g. 500 mg m$^{-2}$ day$^{-1}$) seem to result in increased toxicity, as seen by Spicer *et al.* (1988). What has not been looked at in depth is whether the approach of a short break in treatment every few weeks [as used by Jabboury *et al.* (1989*a*) and Lokich *et al.* (1993)] would allow higher doses or perhaps the same responses but with much less toxicity. If one could thus significantly reduce the mucocutaneous toxicity, but without altering the clinical response, it would make the treatment ideal for palliation, particularly as Chang *et al.* (1989) have shown that it can overcome a lack of response to bolus 5-FU.

Combination chemotherapy is the commonest approach for metastatic disease. It would therefore be very interesting to look in detail at the role of infusional 5-FU in a traditional regimen, such as CAF or CMF, bearing in mind the high response rates noted in Table II. Obviously what is needed is a comparison, and we are currently undertaking such a randomised study to look at the role of continuous 5-FU within CMF for metastatic breast cancer.

The Marsden group have extended their use of ECF (Smith *et al.*, 1993) to the truly adjuvant situation. This is an interesting development for continuous 5-FU with combination chemotherapy. For patients with poor-prognosis breast cancer at presentation, largely as defined by having >10 positive axillary nodes, it would be interesting to examine the role of regimens such as the Marsden's ECF and our AcF, possibly even as an induction prior to high-dose chemotherapy with stem cell rescue (Peters *et al.*, 1993). It is, however, probably premature to challenge the gold standard of adjuvant CMF (Henderson & Shapiro, 1991) for those with lower numbers of axillary nodes.

### References

ANSFIELD, F., RAMIREZ, G., MACKMAN, S., BRYAN, G.T. & CURRERI, A.R. (1969). A 10-year study of 5-fluorouracil in disseminated breast cancer with clinical results and survival times. *Cancer Res.*, **29**, 1062–1066.

BERLIE, J., MEEUS, L., MEYER, C. & ROUESSE, J. (1990). Protracted continuous infusion of 5-fluorouracil among breast cancer patients (abstract 153). *Proc. ASCO*, **9**, 40.

BEVERIDGE, R.A., KALES, A.N., BINDER, R.A., MILLER, J.A. & VIRTS, S.G. (1990). Pyridoxine (B6) and amelioration of hand/foot syndrome (abstract 393). *Proc. ASCO*, **9**, 102.

BONNADONNA, G. & VALAGUSSA, P. (1989). Systemic therapy in resectable breast cancer. In *Hematology/Oncology Clinics of North America: Diagnosis and Therapy of Breast Cancer*, Henderson, I.E. (ed.) pp. 727–742. W.B. Saunders: Philadelphia.

CABALLERO, G.A., AUSMAN, R.K. & QUEBBEMAN, E.J. (1985). Long-term, ambulatory, continuous IV infusions of 5-FU for the treatment of advanced adenocarcinomas. *Cancer Treat. Rep.*, **69**, 13–15.

CARTER, S.K. (1976). Integration of chemotherapy into combined modality treatment of solid tumours. *Cancer Treat. Rev.*, **3**, 141–174.

CHANG, A.Y.C., MOST, C. & PANDYA, K.J. (1989). Continuous intravenous infusion of 5-fluorouracil in the treatment of refractory breast cancer. *Am. J. Clin. Oncol.*, **12**, 453–455.

COHEN, J.L., IRWIN, L.W., MARSHALL, O.J., DARVEY, H. & BATEMAN, J.R. (1974). Clinical pharmacology of oral and intravenous 5-fluorouracil (NSC-19893). *Cancer Chemother. Rep.*, **58**, 723–731.

COLLINS, J.M., DEDRICK, R.L., KING, F.G., SPEYER, J.L. & MYERS, C.E. (1980). Non-linear pharmacokinetic models for 5-fluorouracil in man. *Clin. Pharmacol. Ther.*, **28**, 235–246.

CURRERI, A., ANSFIELD, F., McIVER, F., WAISMAN, H.A. & HEIDELBERGER, C. (1985). Clinical studies with 5-fluorouracil. *Cancer Res.*, **18**, 478–484.

ENSMINGER, W.D., ROSOWSKY, A., RASO, V., LEVIN, D.C., GLODE, M., COME, S., STEELE, G. & FREI III, E. (1978). A clinical pharmacologic evaluation of hepatic arterial infusion of 5-fluoro-2'-deoxyuridine and 5-fluorouracil. *Cancer Res.*, **38**, 3784-3792.

ETIENNE, M.C., MILANO, G., FLEMING, R.A., THYSS, A., RENEE, N., SCHNEIDER, M. & DEMARD, F. (1992). Dihydropyrimidine dehydrogenase activity in lymphocytes: predictive factor for 5-fluorouracil clearance. *Bull. Du Cancer*, **79**, 1159-1163.

FINDLAY, M. & CUNNINGHAM, D. (1993). Chemotherapy of carcinoma of the stomach. *Cancer Treat. Rev.*, **19**, 29-44.

FISHER, B., GUNDUZ, N. & SAFFER, E.A. (1983). Influence of the interval between primary tumour removal and chemotherapy on kinetics and growth of metastases. *Cancer Res.*, **43**, 1488-1492.

FRAILE, R.J., BAKER, L.H., BUROKER, T.R., HORWITZ, J. & VAITKEVICIUS, V.K. (1980). Pharmacokinetics of 5-fluorouracil administered orally by rapid intravenous and by slow infusion. *Cancer Res.*, **40**, 2223-2228.

GABRA, H., CAMERON, D.A., LEE, L. & LEONARD, R.C.F. (1993). Weekly adriamycin and continuous 5-fluorouracil for metastatic and locally relapsed breast cancer – preliminary results (abstract P59). *Br. J. Cancer*, **67** (Suppl. XX), 46.

GORDON, C.J. & BAKER, L.H. (1990). Breast cancer. In *Cancer Chemotherapy by Infusion*, 2nd edn, Lokich, J.J. (ed.) pp. 358-371. Precept Press: Chicago.

GORDON, C.J., VALDIVIESO, M., MARTINO, S., REDMAN, B.G., FLAHERTY, L. & BAKER, L.H. (1990). Continuous intravenous 5-fluorouracil (5-FU) infusion, weekly adriamycin (ADR) and oral cyclophosphamide (CTX) [FAC-CI] in the treatment of metastatic breast carcinoma (MBC) (abstract 200). *Proc. ASCO*, **9**, 52.

GREGORY, W.M., SMITH, P., RICHARDS, M.A., TWELVES, C.J., KNIGHT, R.K. & RUBENS, R.D. (1993). Chemotherapy of advanced breast cancer: outcome and prognostic factors. *Br. J. Cancer*, **68**, 988-995.

GREM, J.L. (1990). Fluorinated pyrimidines. In *Cancer Chemotherapy: Principles and Practise*, Chabner, B.A. (ed.). pp. 180-224. J.B. Lippincott: Philadelphia.

GREM, J.L., HOTH, D.F., HAMILTON, J.M., KING, S.A. & LEYLAND-JONES, B. (1987). Overview of current status and future direction of clinical trials with 5-fluorouracil in combination with folinic acid. *Cancer Treat. Rep.*, **71**, 1249-1264.

GYORGY, P. & ECKHARCK, R.E. (1939). Vitamin $B_6$ and skin lesions in rat. *Nature*, **144**, 512.

HANSEN, R., QUEBBEMAN, E., BEATTY, P., RITCH, P., ANDERSON, T., JENKINS, D., FRICK, J. & AUSMAN, R. (1987). Continuous 5 fluorouracil in refractory carcinoma of the breast. *Breast Cancer Res. Treat.*, **10**, 145-149.

HARRIS, B.E., CARPENTER, J.T. & DIASIO, R.B. (1991). Severe 5-fluorouracil toxicity secondary to dihydropyrimidine dehydrogenase deficiency. *Cancer*, **68**, 499-501.

HATFIELD, A.K., JOHNSON, P.A., EGNER, J.R. & LONG, C.A. (1989). Long term continuous 5-fluorouracil (5-FU) influsion in the treatment of far advanced metastatic breast carcinoma (abstract 132). *Proc. ASCO*, **8**, 35.

HEIDELBERGER, C., CHAUDHUARI, N.K., DANENBERG, P., MOOREN, D. & GREISBACH, L. (1957). Fluorinated pyrimidines: a new class of tumour inhibitory compounds. *Nature*, **179**, 663-666.

HENDERSON, I.C. & SHAPIRO, C.L. (1991). Adjuvant chemotherapy: an overview. In *Medical Management of Breast Cancer*, Powles, T.J. & Smith, I.E. (eds). pp. 197-215. Martin Dunitz: London.

IVESON, T.J., RAMAGE, F.R., WALSH, G., JONES, A.L. & SMITH, I.E. (1993). Preliminary report on continuous infusional 5-fluorouracil with epirubicin and cisplatin in the treatment of locally advanced metastatic breast cancer. *J. Infus. Chemother.*, **3**, 19- 21.

HUAN, S., PAZDUR, R., SINGHAKOWINTA, A., SAMAL, B. & VAIKEVICIUS, V. (1989). Low-dose continuous infusion 5-fluorouracil: evaluation in advanced breast cancer. *Cancer*, **63**, 419-422.

JABBOURY, K., HOLMES, F.A. & HORTOBAGYI, G. (1989*a*). 5-Fluorouracil rechallenge by protracted infusion in refractory breast cancer. *Cancer*, **64**, 793-797.

JABBOURY, K., HOLMES, F., KAU, S. & HORTOBAGYI, G. (1989*b*). Folinic acid modulation of low-dose fluorouacil (FU) infusion in refractory breast cancer: a dose optimization study (abstract 152). *Proc. ASCO*, **8**, 40.

JONES, R.B., SHPALL, E.J., SHOGAN, J., AFFRONTI, M.L., CONIGLIO, D., HART, L., HALPERIN, E., IGLEHART, J.D., MOORE, J., GOCKERMAN, J., BAST, R.C. & PETERS, W.P. (1990). The Duke AFM program: intensive induction chemotherapy for metastatic breast cancer. *Cancer*, **66**, 431-436.

LEMON, H.M. (1960). Reduction of 5-fluorouracil toxicity in man with retention of anticancer effects by prolonged intravenous administration in 5% dextrose. *Cancer Chemother. Rep.*, **8**, 97-101.

LOKICH, J.J. & ANDERSON, N. (1993). Infusional chemotherapy for breast cancer: the Cancer Center of Boston. *J. Infus. Chemother.*, **3**, 9-14.

LOKICH, J.J. & MOORE, C. (1984). Chemotherapy associated palmar-plantar erythrodysesthesia syndrome. *Ann. Intern. Med.*, **101**, 798-800.

LOKICH, J., BOTHE, A., FINE, N. & PERRI, J. (1981). Phase I study of protracted venous infusion of 5-fluorouracil. *Cancer*, **48**, 2565-2568.

LOKICH, J., PHILLIPS, D., GREEN, R., PAUL, S., SONNEBURN, H., ZIPOLI, T.E. & CURT, G. (1985). 5-Fluorouracil and methotrexate administered simultaneously as a continuous infusion: a phase I study. *Cancer*, **56**, 2395-2398.

LOKICH, J.J., AHLGREN, J.D., GULLO, J., PHILLIPS, J.A. & FRYER, J.G. (1989). A prospective randomised comparison of continuous infusion fluorouracil with a conventional bolus schedule in metastatic colorectal carcinoma: a Mid-Atlantic Oncology Program Study. *J. Clin. Oncol.*, **7**, 425-432.

MORTIMER, J.E. & ANDERSON, I. (1990). Weekly fluorouracil and high-dose leucovorin: efficacy and treatment of toxicity. *Cancer Chemother. Pharmacol.*, **26**, 449-452.

NG, J., CAMERON, D.A., LEE, L., DIXON, J.M. & LEONARD, R.C.F. (1994). Infusional 5-fluorouracil given as a single agent in relapsed breast cancer: its activity and toxicity. *Breast* (in press).

O'BRIEN, M.E., SMITH, I.E., TALBOT, D.C., BAUM, M., SACKS, N. & MCKINNA, J.A. (1992). Long-term continuous infusion of 5-FU (F) with epirubicin (E) and cisplatin (C) in the treatment of breast cancer: a very active new regimen (abstract 120). *Proc. ASCO*, **11**, 73.

PETERS, W.P., ROSS, M., VREDENBURGH, J.J., MEISENBURG, B., MARKS, L.B., WINER, E., KURTZBERG, J., BAST, R.C. Jr, JONES, R., SHPALL, E., WU, K., ROSNER, G., GILBERT, C., MATHIAS, B., CONIGLIO, D., PETOR, W., HENDERSON, C., NORTON, L., WEISS, R.B., BUDMAN, D. & HURD, D. (1993). High-dose chemotherapy and autologous bone marrow support as consolidation after standard-dose adjuvant therapy for high-risk primary breast cancer. *J. Clin. Oncol.*, **11**, 1132-1143.

PINEDO, H.M. & PETERS, G.F.J. (1988). Fluorouracil: biochemistry and pharmacology. *J. Clin. Oncol.*, **6**, 1653-1664.

RUBENS, R.D. (1991). Single agent chemotherapy. In *Medical Management of Breast Cancer*, Powles, T.J. & Smith, I.E. (eds). pp. 133-137. Martin Dunitz: London.

SAPHNER, T., TORMEY, D.C., ALBERTINI, M. & WINOKUR, S. (1991). Phase I trial of continuous infusion 5-FU with weekly bolus cis-platinum and etoposide (abstract 92). *Proc. ASCO*, **10**, 55.

SMITH, I.E., JONES, A.L., WALSH, G., BAUM, M., EBBS, S. & SACKS, N. (1993). Primary medical chemotherapy with continuous infusional 5-fu (F), epirubicin (E) and cisplatin (P) for large operable breast cancer: a very active new regimen (abstract 12). *Breast*, **2**, 188.

SPICER, D.V., ARDALAN, B., DANIELS, J.R., SILBERMAN, H. & JOHNSON, K. (1988). Re-evaluation of the maximum tolerated dose of continuous venous infusion of 5-fluorouracil with pharmacokinetics. *Cancer Res.*, **48**, 459-461.

STRAUSS, G., SCHWARTZ, J., NICKESON, J. & LOKICH, J. (1988). Continuous infusion (CI) 5-fluorouracil (5-FU) with or without cisplatinum as 3rd line therapy for metastatic breast cancer (abstract 153). *Proc. ASCO*, **7**, 39.

SULLIVAN, R.D., YOUNG, C.W., MILLER, E., GLATSTEIN, N., CLARKSON, B. & BURCHENAL, J.H. (1960). The clinical effects of the continuous administration of fluorinated pyrimidines (5-fluorouracil and 5-fluoro-2'-deoxyuridine). *Cancer Chemother. Rep.*, **8**, 77-81.

TEMPERO, M., MITCHELL, M. & HIGGINBOTHAM, P. (1991). Tolerance of protracted infusion 5-fluorouracil and oral leucovorin (abstract 505). *Proc ASCO*, **10**, 160.

VAN HOEF, M.E.H.M., ZONNENBERG, B.A., DE GRAEFF, A., VAN MILLIGEN DE WIT, A.W.M., TJIA, P. & NEIJT, J.P. (1991). Ambulante continue intraveneuze infusie van fluorouracil: een haalbare palliatieve chemotherapie. *Ned. Tijdschr. Geneeskd.*, **135**, 563-567.

VOGELZANG, N.J. (1984). Continuous infusion chemotherapy: a critical review. *J. Clin. Oncol.*, **2**, 1289-1304.

WOODCOCK, T.M., MARTIN, D.S. & DAMIN, L.E.M. (1980). Clinical trials with thymidine and fluorouracil: a phase I and clinical pharmacological evaluation. *Cancer*, **45**, 1135-1143.